# Wnt/β-Catenin Pathway and Hydraulic Calcium Silicate-Based Cements: A Narrative Review

**DOI:** 10.3390/dj14050273

**Published:** 2026-05-06

**Authors:** Carmela Del Giudice, Carmen Vito, Gianrico Spagnuolo, Carlo Rengo, Alessandra Valletta, Ciro Menale, Flavia Iaculli

**Affiliations:** 1 Department of Neuroscience, Reproductive Science and Dentistry, Federico II University of Naples, 80131 Naples, Italy; carmela.delgiudice@unina.it (C.D.G.); carmen.vito@unina.it (C.V.); alessandra.valletta@unina.it (A.V.); 2 Department of Medicine, Surgery and Dentistry, University of Salerno, 84081 Baronissi, Italy; crengo@unisa.it; 3 Department of Clinical Medicine and Surgery, Federico II University of Naples, 80131 Naples, Italy; 4 Department of Oral and Maxillofacial Sciences, Sapienza University of Rome, 00161 Rome, Italy; flavia.iaculli@uniroma1.it

**Keywords:** dentinal–pulp complex, hydraulic cements, regeneration, signaling pathway, tissue repair, Wnt/β-catenin

## Abstract

The Wnt/β-catenin signaling pathway regulates key cellular processes, including proliferation, migration, differentiation, apoptosis and tissue homeostasis, and plays a pivotal role in tooth development and post-developmental dental physiology. In mineralized tissues such as bone and dentin, the Wnt signaling is critically involved in reparative and regenerative mechanisms. The Wnt signaling in the dentin–pulp complex is tightly controlled by extracellular modulators and receptor availability, and its balance appears crucial for an appropriate response. Hydraulic calcium silicate-based cements (HCSBCs) are widely used in endodontics due to their bioactivity and favorable biological properties. Increasing data indicate that HCSBCs promote odontogenic responses and reparative dentinogenesis through the recruitment and activation of dental stem cells (DSCs), possibly via the Wnt/β-catenin signaling pathway modulation. Therefore, the aim of the present narrative review was to summarize current knowledge on the role of the Wnt signaling in oral tissues and its interaction with HCSBCs. It is hypothesized that these materials may enhance pathway activation through the release of ionic products, growth factors and inflammatory mediators, thereby supporting biologically driven reparative processes. Understanding these mechanisms may guide the development of next-generation biomaterials designed to optimize the intrinsic regenerative potential of the dentin–pulp complex.

## 1. Introduction

The Wnt/β-catenin signaling pathway participates in various physiological processes such as cell migration, differentiation, proliferation, invasion, apoptosis and tissue homeostasis [1,2]. This pathway plays a critical role in dental biology by influencing regenerative processes, especially in mineralized tissues such as bone and teeth [3]. Its function is regulated through the activation and inhibition of various molecular mechanisms. Among these, the Wnt signaling is an evolutionarily conserved pathway that is crucial for both tooth development and post-developmental tooth physiology [4].

Dental materials are widely used in restorative procedures, implant dentistry and orthodontic applications, and their biocompatibility and efficacy are essential for successful clinical outcomes. Recent studies support the hypothesis that cellular response to dental materials may be mediated by activation of the Wnt/β-catenin signaling pathway [5,6,7], which has been shown to influence several cellular behaviors [8]. Disruptions or alterations in the Wnt signaling can lead to impaired tissue regeneration and might contribute to complications in dental treatments.

Hydraulic calcium silicate-based cements (HCSBCs) are currently and successfully applied in endodontic field due to their biocompatibility, bioactivity and great biological properties, e.g., stimulation of mineralized tissue deposition and sealing ability [9]. These cements are largely considered as gold standard materials in several treatments involving the dentin–pulp complex such as [10] vital pulp therapy, perforation repair, apical closure and regenerative procedures [11,12,13].

HCSBCs exert a significant influence on cellular stimulation in an odontogenic pattern, thereby enhancing reparative dentinogenesis via the recruitment and activation of hard tissue-forming cells [10]. Wnt/β-catenin signaling pathways have been suggested to play key roles in mediating the biological interactions between dental stem cells (DSCs) and HCSBCs. Elucidating the signaling mechanisms underlying these interactions may enable the design of innovative biomaterial formulations aimed at promoting tissue repair through biologically driven approaches [14]. Therefore, the aim of the present review was to report the effect of Wnt/β-catenin signaling pathway on oral tissues and in response to HCSBCs. The hypothesis is that the presence of HCSBCs induces the release of ionic products, growth factors and inflammatory mediators, resulting in enhanced activation of the Wnt/β-catenin signaling pathway.

## 2. Wnt/β-Catenin: Molecular Mechanism

Wnts are one of the major family of signaling molecules that influence biological processes. The signal transduction mechanism of this pathway provides the binding of Wnt ligand to two receptors [15]: the seven-pass transmembrane Frizzled (Fz or Fzd) receptor and the low-density lipoprotein receptor-related protein 5 or 6 (LRP5/6), both localized on cell surface. Upon the Wnt signaling activation, disheveled (DVL) is recruited to the receptor complex (Fzd and LRP5/6), where it functions as a mediator protein by helping the recruitment of Axin and associated components (GSK3β, CK1, and APC), thereby promoting complex formation. Subsequently, the phosphorylation and inhibition of GSK3β ensure an elevation of un-phosphorylated β-catenin concentration in the cytosol that migrates to the nucleus, interacting with T cell-specific factor (TCF)/lymphoid enhancer-binding factor (LEF) and activating Wnt target gene expression [16,17]. In the Wnt/β-catenin signaling pathway, abnormal regulation of transcription factor β-catenin leads to early dysfunctional events. Within the degradation complex, in absence of Wnt ligand, glycogen synthase kinase 3β (GSK3β) and casein kinase 1α (CK1α) mediate the phosphorylation of β-catenin, promoting its degradation [18]. Recently, an in vivo study conducted on Drosophila regarding dynamic Wnt signal transduction, confirmed that β-catenin degradation was influenced by modified Axin-GSK3β interactions, caused by conformational change within the destruction complex [19]. In addition to the canonical Wnt/β-catenin signaling pathway, Wnt ligands can also initiate a group of β-catenin-independent signaling cascades collectively referred to as non-canonical pathways [20]. These alternative routes play critical roles in regulating cytoskeletal dynamics, intracellular calcium signaling, and planar cell polarity, processes essential for tissue morphogenesis and cell migration. Two primary branches of the non-canonical Wnt signaling have been described:

- The Wnt/planar cell polarity (PCP) pathway, which is vital for coordinating cell polarity, directed migration, and tissues organization during development.
- The Wnt/Ca^2+^ pathway, which modulates intracellular calcium levels and activates downstream effectors involved in cell motility, adhesion and other calcium-dependent processes.

Unlike canonical signaling, these non-canonical pathways do not rely on β-catenin stabilization or nuclear translocation, but engage distinct molecular machinery to elicit context-specific cellular responses. In the context of oral and dental tissues, the Wnt/β-catenin signaling pathway plays a pivotal role in tooth development, tissue regeneration and oral stem cell regulation [21,22].

## 3. Activators and Inhibitors of the Pathway

Within the dentin–pulp complex, the Wnt/β-catenin signaling pathway is regulated by extracellular modulators and receptor availability. This aspect may be particularly relevant to the reparative and regenerative capacity of dental tissues, and the use of HCSBCs could potentially shift the balance between Wnt activators and inhibitors. Various molecules influence the Wnt signaling function. Some regulators, such as glypicans, R-spondin, casein kinase and norrin can activate the pathway.

Glypicans are membrane-bound heparan sulfate proteoglycans that play a critical role in modulating the extracellular distribution and activity of Wnt proteins [8]. Particularly, Glypican-3 (GPC3) is thought to interact with the central region of Wnt3a through its cysteine-rich domain (CRD). This interaction enhances Wnt signaling, especially under conditions in which Fzd receptors are highly expressed. The synergistic activation of the Wnt signaling is primarily mediated by the binding of GPC3 to Fzd via its heparan sulphate (HS) chains, facilitating the coordinated regulation of the pathway [23,24]. McLachlan et al. in 2005 have highlighted the differential expression of several genes in dental pulp tissue affected by dental caries, even including GPC3—a gene not previously linked to this condition [25]. However, the precise role of GPC3 in dental biology remains unclear, and its potential involvement in processes such as osteogenesis, bone remodeling and enamel formation should be further elucidated.

R-spondins (Rspos) are potent agonists of the Wnt signaling [26]. The Rspos protein family consists of four members (Rspos 1–4), which share similar structural domains [27]. Rspos promote the Wnt signaling by inducing the binding of the LGR4/5 receptors to the negative feedback regulators Znrf3/Rnf43, which are E3 ubiquitin ligases associated with the degradation of Fzd receptors [28]. By facilitating the removal of these ligases from the cell membrane, Rspos reduce the ubiquitination of Fzd receptors, thereby enhancing Wnt/β-catenin signal transduction [29]. In recent years, increasing attention has been committed to understanding the roles and mechanisms of Rspos in several biological processes, including bone development and metabolism. Sharma et al. [30] firstly reported a positive regulatory role for Rspo1 in osteogenic differentiation. In both human primary osteoblasts and the pre-osteoblastic cell line FOB1.19, an increase in Rspo1 expression was observed during differentiation. Administration of recombinant Rspo1 enhanced alkaline phosphatase (ALP) activity and promoted osteogenesis through activation of the Wnt/β-catenin signaling pathway [30]. Similarly, Rspo2 has been implicated in promoting osteogenic differentiation and bone formation [31]. Despite these promising findings, many aspects of Rspo signaling in dental development remain unclear.

Members of Casein Kinase (CK) also participate in the Wnt signaling by phosphorylating various components, including the Wnt receptor complex and Axin [32]. CK1 can aid in the stabilization of the signalosome and the activation of downstream signaling [32,33,34]. While no studies have reported positive effects on osteogenesis or dental development, in 2020, Tian et al. identified CKIIP-1 (Casein Kinase 2-Interacting Protein-1) as a negative regulator of mesenchymal stem cell (MSC) osteogenesis, exhibiting age-dependent effects [35].

Norrin protein is a signaling molecule which can also bind Fzd class receptor 4 (Fzd4) and LRP5/6 to form a ternary complex and promote the downstream signal transmission of Wnt/β-catenin [36,37]. It plays a role in retinal development and in vascular signaling [38]. Although Norrin effectively activates the canonical Wnt/β-catenin signaling pathway, its direct contribution to dental development and oral tissues needs to be directly demonstrated.

On the other hand, various proteins can antagonize and modulate the Wnt/β-catenin signaling pathway. Several regulatory mechanisms have been related to pathway destiny, such as Dickkopf (DKK), some of the Secreted Frizzled Related-Proteins family (sFRPs), Wnt inhibitory factor (WIF), cardamonin and Sclerostin (SOST), which influences bone formation processes.

DKK is a family of protein of four members (Dkk1–4) secreted with two cysteine-rich regions. They are implicated in embryonic development through negative regulation of the Wnt/β-catenin signaling pathway by interacting to LRP6 receptors (preventing Fz-LRP6 complex formation) [39] or leading to endocytosis of LRP6 [40], resulting in blocking of Wnt binding and β-catenin degradation [41]. In the absence of Wnt, destruction complex binds β-catenin, which is phosphorylated by GSK3; on turn, phosphorylation triggers ubiquitin-mediated degradation [16]. It has been reported that Dkks have a key role in human development, and, in adults, they inhibit the pathways implicated in regulation of bone formation [42].

The five members of the sFRP family are soluble proteins that play an important function in inhibiting the Wnt/β-catenin signaling pathway [43,44]. Maekawa et al. highlighted the role of the antagonist sFRP5 in periodontal health and disease [45]. In human gingival tissues, sFRP5 expression was significantly higher in healthy samples compared to those affected by periodontitis. Furthermore, in vivo administration of sFRP5 in a mouse model of ligature-induced periodontitis reduced inflammation, osteoclast numbers and bone loss, further supporting its protective role [45]. Accordingly, Yamada et al. suggested that in human mesenchymal stem cells (hMSCs), sFRPs modulated osteoblastogenesis in opposing manner through canonical and the non-canonical Wnt signaling pathways, with sFRP3 promoting and sFRP4 inhibiting osteogenic differentiation of hMSCs [46].

WIF structure has an N-terminal signal sequence, a Wnt ligand binding domain, five epidermal growth factor-like repeats and a short hydrophilic domain [47,48]. As evidence of signaling inhibition, WIF has shown to significantly impair Wnt3a-dependent transcriptional activity of TCF⁄LEF [49] and should be part of the Wnt signaling negative feedback control [50]. Consistently, Cho et al. demonstrated that WIF-1 functioned as a negative regulator of osteoblastic differentiation in mouse embryonic mesenchymal cells, further supporting its inhibitory influence on Wnt-driven osteogenesis [51].

In the same way, cardamonin—a chalcone that belongs to the flavonoid family, isolated from different plants—inhibits the biological activity of Wnt/β-catenin [52]. In 2024, a study conducted by Meng et al. investigated the therapeutic effects and potential mechanism of cardamonin in the regulation of subchondral osteosclerosis associated with osteoarthritis, with both models using a mouse calvarial pre-osteoblast cell line [53]. Their findings suggested that cardamonin may exert its bone-modulating effects, at least in part, through inhibition of the Wnt/β-catenin signaling pathway [53].

SOST is mainly expressed in osteocytes and in osteoblasts, and loss of function mutations or downregulation lead to diseases characterized by bone tissue overgrowth [54]. Like human, SOST-null mutant mice have a high-bone mass phenotype associated with increase in bone mass density, volume, formation and strength [55]. SOST antagonizes the Wnt signaling by binding the domains of LRP5 and LRP6 [56,57]. Probably, the loss of SOST function leads to the hyperactivation of the Wnt signaling causing bone overgrowth [58].

Together, these Wnt signaling regulators orchestrate a finely tuned balance between activation and inhibition, which is critical for maintaining tissue homeostasis. In the context of oral and dental biology, this balance governs processes such as enamel formation, periodontal ligament regeneration, alveolar bone remodeling and response to inflammation or injury (Table 1). Moreover, the complex network of Wnt/β catenin modulators provides a finely tuned regulatory system, which might help explain the tissue-specific reparative responses to bioactive materials.

## 4. Wnt Correlation with Dental Development

Genetic studies have demonstrated that the Wnt/β-catenin signaling pathway is of key importance during tooth formation [59]. Throughout tooth initiation phase and morphogenesis, some Wnts signaling molecules such as Wnt3, Wnt7b, Wnt10a and Wnt10b regulate key cellular processes [60]. During the bug and cap stages, the Wnt signaling continues to be active in epithelial and mesenchymal cells [61]. Liu et al. also demonstrated that when the Wnt/β-catenin signaling pathway was inhibited by epithelial expression of secreted DKK1, dental development was arrested at the bug stage [61]. Moreover, at the bell stage, the Wnt/β-catenin signaling pathway is localized in the secondary enamel knots and in the underlying mesenchymal cells [62]. Furthermore, modulation of this pathway plays an important role in the reparative dentine formation following tooth damage in mice molars, by triggering the natural process of dentinogenesis [63]. Although during pulp repair Transforming Growth Factor-β1 (TGF-β1) seems to be associated with the Wnt/β-catenin signaling pathway to promote diverse biological effects [64], the molecular mechanisms of inactive odontoblasts activation, pulp stem cells stimulation and tertiary dentin formation after stimulation with restorative materials are still unclear [65]. In this light, Yoshioka et al. reported an increase of β-catenin in pulp cells under cavities, indicating that the Wnt/β-catenin signaling pathway might be activated by cavity preparation [65]. Moreover, it has been demonstrated that inhibition of the Wnt/β-catenin signaling pathway is important for dentin and cementum formation during tooth development and may be therapeutically exploited through local modulation to enhance dentin and periodontium regeneration [66].

Remodeling processes involving bone formation and resorption around the tooth root take place during postnatal life as well as during orthodontic treatment. In response to mechanical forces, it is still uncertain whether Wnt/β-catenin signaling pathway, a key regulator of bone maintenance, participates in mechanotransduction in the alveolar bone or in periodontal ligament activities. In this light, Liu et al. conducted a study using periodontal ligament stem cells (PLSCs) and observed that the inhibition of the Wnt signaling seemed to regulate osteogenic differentiation in inflammatory microenvironments [67].

A thorough understanding of the context-dependent modulation of the Wnt signaling is therefore essential, not only for advancing targeted regenerative strategies but also for enhancing the biofunctional performance of dental materials. Specifically, elucidating how specific oral cell populations respond to Wnt modulation is crucial for rationalizing the biological effects of HCSBCs.

## 5. Wnt Signaling in the Oral Cells

The Wnt/β-catenin signaling pathway exerts cell-type-specific effects in oral tissues. In 2008, modulation of odontoblast-like differentiation of dental pulp stem cells (DPSCs) by Wnt/β-catenin signaling pathway was evaluated [68]. The Authors observed that Wnt-1 inhibited alkaline phosphatase (ALP) activity and mineralized nodule formation in DPSCs, while inducing β-catenin overexpression, which in turn suppressed DPSCs differentiation and mineralization [68]. These results demonstrated that the Wnt/β-catenin signaling pathway negatively regulates odontoblast-like differentiation of DPSCs [68]. On the other hand, Bakopoulou and co-authors reported that the Wnt signaling played a significant role in DPSCs destiny, promoting proliferation and differentiation [5]. They demonstrated that Wnt cascade could be activated in DPSCs after Lithium or Wnt-1 treatment, resulting in pathway activation and target gene expression [5]. The apparently contradictory effects observed—despite the use of the same Wnt-1 ligand—may be attributed to variations in exposure duration, differences in the stage of DPSCs commitment, microenvironmental conditions, or the presence or absence of resin monomer treatment. In 2009, Nemoto et al. studied the correlation between the Wnt signaling and cementoblasts, demonstrating that the Wnt/β-catenin signaling pathway repressed their differentiation, regulating the expression of transcription factors, such as ALP, BSP, and OCN gene and observed that Wnt3a favored the expression of cyclin D1, stimulating cell proliferation. These outcomes suggested that the Wnt/β-catenin signaling pathway might prevent cementoblast differentiation and stimulate cell proliferation [69]. Accordingly, Zhou et al. evaluated the involvement of the Wnt/β-catenin signaling pathway to determinate proliferation and cementogenic differentiation of periodontal ligament cells when in contact with extracts derived from bioactive bredigite (Ca_7_MgSi_4_O_16_) bioceramics. The authors showed that extracts promoted proliferation and cementogenic differentiation and demonstrated that the addition of cardamonin reduced the pro-cementogenesis effect of the bredigite powder extracts [70].

In 2012, Wang et al. stimulated stem cells from the apical papilla (SCAPs), in presence or absence of various concentrations of lithium chloride, showing that canonical Wnt/β-catenin signaling pathway encouraged proliferation and odonto/osteogenic differentiation of SCAPs [71]. Few years later, the effect of STRO-1, one of the most common mesenchymal stem cell markers, and Wnt3a treatment on human periodontal ligament-derived progenitor cells (hPDLCs) was investigated [72]. It has been demonstrated that Wnt3a supported hPDLC proliferation and preserved self-renewal and osteogenic differentiation ability [72]. As previously reported, in periodontitis, the Wnt/β-catenin signaling pathway plays an important role in the regeneration of alveolar bone [73]. Indeed, Liu et al. used human gingival fibroblast (HGF) to study the interaction between the Wnt/β-catenin signaling pathway and the extracellular matrix metalloproteinases inducer expression correlated with matrix metalloproteinases (EMMPRIN/MMPs) in periodontitis [74]. They observed that the aberrant activation of the Wnt signaling impeded the EMMPRIN/MMP-2, 9 routes and the block of EMMPRIN reducing the Wnt/β-catenin signaling pathway [74].

Despite these findings, the role of the Wnt/β-catenin signaling pathway in odonto/osteogenic differentiation remains controversial, as both stimulatory and inhibitory effects have been reported. While several studies demonstrated that activation of Wnt/β-catenin signaling promotes proliferation and odonto-/osteogenic differentiation of DSCs [5], other investigations showed that excessive or prolonged activation may inhibit mineralization and odontoblast-like differentiation [68]. This apparent discrepancy suggests that the Wnt signaling does not exert a unidirectional effect, but rather acts as a context-dependent regulator of cell fate. Several factors may account for these conflicting findings. Indeed, differences in cell type (e.g., DPSCs, SCAPs, periodontal ligament cells, or cementoblasts) may significantly influence the cellular response to Wnt activation, as each population exhibits distinct differentiation potential and receptor expression profiles. Furthermore, experimental conditions, as the type of Wnt ligand used (e.g., Wnt1 vs. Wnt3a) and the presence of co-stimulatory or inhibitory molecules, may alter pathway dynamics. Finally, the timing of treatments appears critical: early activation may favor proliferation and maintenance of stemness, whereas sustained activation may impair terminal differentiation and mineral deposition. In addition, dose-dependent effects of pathway modulators, such as lithium chloride or biomaterial-derived extracts, may further contribute to heterogeneous outcomes.

Despite extensive research on the canonical Wnt/β-catenin signaling pathway, the involvement of the non-canonical Wnt signaling—that may regulate oral cell behavior particularly in response to inflammatory or mechanical stimuli—remains poorly characterized. In periodontal tissues, Wnt5a contributes to ligament development and maintenance, modulates bone homeostasis, and is upregulated during periodontitis, suggesting a multifaceted role in both homeostasis and inflammation [75]. Furthermore, under tensile forces experienced by periodontal ligament cells, activation of a YAP–Wnt5a–Fzd4 axis promotes osteogenic differentiation [76], while in mechanically stimulated bone cells, Wnt5a-Ror2 signaling engages RhoA-driven cytoskeletal remodeling to promote osteogenesis [77]. However, the specific contributions and regulatory mechanisms of non-canonical versus canonical Wnt pathways in oral cells, especially in varying microenvironments, remain to be fully defined.

## 6. Wnt Signaling and Hydraulic Calcium Silicate-Based Cements

A novel translational approach to the improvement of future clinical dental therapies is grounded in the exploitation of the intrinsic reparative capacity of dental tissues, with particular emphasis on biologically based repair mechanisms mediated by different signaling pathways involved in reparative dentin formation. Within the dental pulp, mesenchyme-derived specialized cells—odontoblasts—are responsible for dentin secretion throughout the lifespan. Given its role in dental repair, it is of considerable interest to underline how this pathway plays a role in reactionary dentin formation across different clinical contexts [22]. In addition, the positive effect of HCSBCs on reparative dentinogenesis has been well documented [10], suggesting a possible effect even on the Wnt/β-catenin signaling pathway. It has been reported that bone morphogenetic proteins (BMPs) [78,79,80,81], fibroblast growth factors (FGFs) [82,83] and Wnts [4,61] may be involved in the repair process by promoting the odonto/osteogenic differentiation of stem/progenitor cells. Nevertheless, the precise mechanisms underlying the role of the Wnt signaling in dentin bridge formation following direct pulp capping remain unclear. Several studies suggest that the Wnt/β-catenin signaling pathway plays a key role in regulating odontoblastic differentiation. Upregulation of β-catenin expression promotes the odontoblastic differentiation of DPSCs, whereas its downregulation or knockdown inhibits cellular differentiation [14]. Furthermore, HCSBCs seem to upregulate a series of signaling transduction pathways (i.e., MAPK, NF-beta, Wnt β-catenin, BMP/Smad and CAMKII) as factors influencing hDPSC differentiation in odonto-/osteogenic pattern [84].

Chen et al. showed that the Wnt/β-catenin signaling pathway-related genes and proteins were significantly expressed when hDPSCs were cultured in a wide concentration range of Mineral Trioxide Aggregate (MTA) extracts (6.25 to 100 mg/mL) compared to control [85] (Table 2). These outcomes supported the activation of the Wnt/β-catenin signaling pathway in presence of MTA, promoting odontogenic differentiation of progenitor cells. Accordingly, involvement of the Wnt/β-catenin signaling pathway following direct pulp capping was observed 14 days after treatment using MTA [86] (Table 2). This was evidenced by the formation of dentine bridge-like calcified tissue beneath the perforation site, along with strong β-catenin expression in odontoblast-like cells and pulp cells in the same area, highlighting the pivotal role of β-catenin in odontoblastic differentiation during reparative dentin formation.

A recent study reported that exposed dental pulps in a rat molar directly capped with calcium hydroxide (Ca(OH)_2_), MTA and Biodentine showed reparative dentine formation near the exposure site, significantly greater in groups treated with hydraulic materials. Moreover, all tested materials induced cyclin D1 expression within pulp tissue; however, only Biodentine showed β-catenin expression in the pulp cells close to the newly formed reparative dentine [87] (Table 2). Similarly, β-catenin expression was only enriched in primary dental pulp cells of Biodentine-treated rat molars when compared to the exposure to MTA or resin-reinforced calcium silicate cement (TheraCal LC). These results suggested that the Wnt/β-catenin signaling pathway may be related to the reparative dentin formation inducted by a specific bioactive material [88] (Table 2).

Activation of the Wnt/β-catenin signaling pathway has also been reported following direct pulp capping with resin-reinforced calcium silicate cement (TheraCal LC). The expression of Wnt3a, Wnt10a, and β-catenin in odontoblasts and dental pulp cells was observed at 7 and 14 days post-treatment. Moreover, at 28 days—when dentin bridge formation was evident—reparative odontoblasts exhibited expression of Wnt3a, β-catenin, and osterix. Additionally, F4/80- and Wnt10a-positive macrophages were identified in the central region of dental pulp. The presence of canonical Wnt ligands further supports the activation of the Wnt signaling in the healing of the dentinal-pulp complex [89].

The involvement of the Wnt/β-catenin signaling pathway is also demonstrated testing a nanoparticle bioceramic material (iRoot FS) used in the obtaining of apical plug of permanent teeth. Indeed, culture of human SCAPs in the presence of iRoot FS revealed significant activation of the Wnt/β-catenin signaling pathway and supported the positive influence on osteo/odontogenic differentiation [90] (Table 2).

**Table 2:** Overview of the effects of different materials on the Wnt signaling modulation and odontogenic responses.

Material	Wnt/β-Catenin Modulation	Odontogenic Response	Study Model	Ref.
MTA	Upregulated	hDPSCs differentiation	In vitro/In vivo	[85,86]
Biodentine	Activated	Reparative dentin formation	In vivo	[87]
TheraCal LC	Activated	Dentin bridge formation	In vitro	[88]
iRoot FS	Activated	Odontogenic differentiation	In vitro	[90]

**Figure 1:**
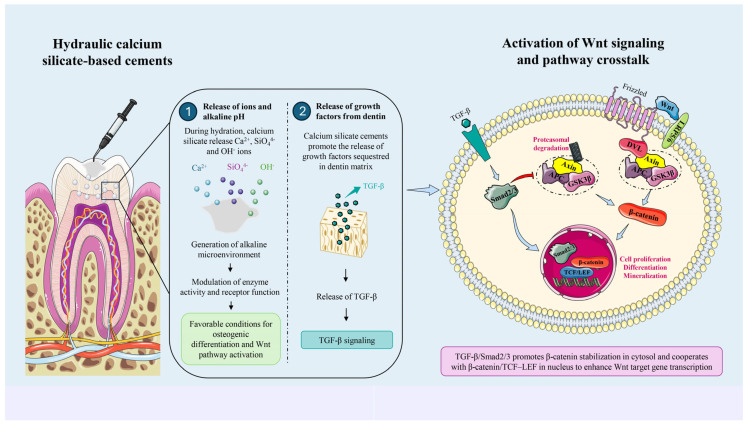
Proposed mechanism by which hydraulic calcium silicate-based cements (HCSBCs) modulate the Wnt/β-catenin signaling pathway. Ion release (Ca^2+^, SiO_4_^4−^, OH^−^) and alkaline pH create a favorable microenvironment, while the release of dentin-derived growth factors, including TGF-β, promotes pathway crosstalk. TGF-β, via Smad2/3 activation, promotes β-catenin accumulation by inhibiting its degradation complex (Axin/APC/GSK3β) and enhances the Wnt signaling through direct interaction with β-catenin/TCF–LEF, leading to transcriptional co-activation of target genes.

Although the use of HCSBCs appears to have a positive impact on the Wnt/β-catenin signaling pathway (Figure 1), the currently available scientific evidence is limited, and the results are preliminary and largely based on in vitro studies. It should also be considered that, as previously described, the pathway activation appears to be context-dependent, and in the presence of HCSBCs it may be differentially modulated depending on several experimental and biological variables, such as variations in cell type, exposure duration, co-treatment conditions and chemical nature of materials, significantly influencing the magnitude and direction of pathway activation. Furthermore, the molecular mechanism supporting the association between HCSBCs and the Wnt/β-catenin signaling pathway remains unclear. Nevertheless, HCSBCs are widely recognized for their biocompatibility and bioactivity, including their ability to release ions and stimulate cellular responses involved in tissue regeneration [91,92,93,94]. Moreover, these materials generate an alkaline microenvironment through the release of hydroxyl ions during hydration, which may influence cellular signaling by modulating enzyme activity and receptor function, thereby creating conditions favorable for osteogenic differentiation and the Wnt signaling activation [95]. In parallel, HCSBCs have been shown to promote the release of growth factors such as TGF-β from the dentin matrix [96], which may further enhance the Wnt signaling through pathway crosstalk [97,98]. Specifically, several studies have demonstrated that TGF-β signaling can modulate the Wnt/β-catenin signaling pathway through multiple mechanisms. In the cytosol, activation of Smad2/3 has been shown to promote β-catenin accumulation by inhibiting its degradation complex, including Axin, APC, and GSK3β. In the nucleus, TGF-β further enhances the Wnt signaling through direct interaction with the β-catenin/TCF–LEF transcriptional complex, thereby facilitating the co-activation of downstream target genes [97,98]. Taken together, these observations provide a biologically plausible—although still indirect—molecular mechanism by which HCSBCs may influence the modulation of the Wnt/β-catenin signaling pathway (Figure 1).

Finally, it should be considered that the interaction between biomaterials and the pathway may also be impaired by the presence of inflammation. In healthy dental pulp, the Wnt/β-catenin signaling pathway contributes to tissue homeostasis by maintaining odontoblast activity and supporting normal cellular turnover. Conversely, in inflamed pulp tissue, the release of inflammatory cytokines (i.e., IL-1β, TNF-α, and IL-6) differently interact with the pathway, either promoting reparative dentinogenesis or impairing tissue repair capacity in cases of acute or chronic inflammation, respectively [99]. Therefore, these aspects should be carefully considered when evaluating the in vivo performance of HCSBCs and their impact on Wnt/β-catenin signaling.

## 7. Future Perspectives and Limitations

The Wnt/β-catenin signaling pathway might be regarded as a molecular interface between HCSBCs-derived cues and the reparative response of the dentin–pulp complex. However, to date, the precise mechanism underlying the pathway enhancement in presence of HCSBCs is not fully elucidated [100]. It has been speculated that the release of calcium ions might have a potential impact in the activation of the canonical Wnt/β-Catenin cascade [101], even though evidence is still lacking. Understanding the interaction between currently used materials and the Wnt/β-catenin signaling pathway has important clinical implications for vital pulp therapy. Widely used materials such as HCSBCs may not only provide a physical barrier and antibacterial environment but also actively modulate cellular responses within the dentin–pulp complex. Identifying materials that more effectively stimulate endogenous reparative pathways may increase treatment predictability, reduce the need for more invasive procedures such as root canal therapy, and improve long-term tooth survival. The growing understanding of molecular mechanisms involved in pulp healing provides an opportunity for the development of next-generation pulp capping materials. Future biomaterials might be specifically engineered to enhance odontogenic responses and reparative dentin formation through targeted modulation of signaling pathways—including the Wnt/β-catenin signaling pathway—incorporating bioactive ions capable of stimulating stem cell recruitment or inducing controlled release of signaling molecules or growth factors. Additionally, tailoring materials might specifically activate endogenous repair mechanisms to reduce inflammatory responses and improve pulp survival, representing a significant advancement in regenerative endodontics and minimally invasive dentistry. Finally, future studies should be focused on the effect of composite restorative materials in the activation/inhibition of the Wnt/β-catenin signaling pathway. In deep cavities, the proximity of composite resins to dental pulp tissue might be considered as prognostic factor for undifferentiated cells recruitment and deposition of a mineralized matrix during reparative dentinogenesis [102]. However, no studies are available on this topic and this aspect remains largely unexplored.

Several limitations currently hinder the translation of these biological insights into routine clinical practice. Most available studies are based on in vitro experiments or animal models, which may not fully replicate the complexity of the human dentin–pulp complex and clinical conditions such as bacterial contamination, trauma and patient-related factors. Consequently, the overall level of clinical evidence remains low to moderate, with a scarcity of well-designed randomized clinical trials and long-term human studies.

## 8. Conclusions

The Wnt/β-catenin signaling pathway plays a central role in tooth development, tissue regeneration and oral stem cell regulation. Moreover, the ability of HCSBCs to release ions, generate an alkaline microenvironment and promote the release of growth factors seem to create a condition favorable for osteogenic differentiation and the Wnt/β-catenin signaling pathway activation. The enhancement of the pathway expression during the reparative response of the dentin–pulp complex might promote biologically driven repair processes, providing the groundwork for the development of next-generation materials designed to optimize the intrinsic regenerative potential of pulp and dentin tissues by activating endogenous repair mechanisms, reducing inflammation, improving pulp survival and advancing regenerative endodontics and minimally invasive dentistry.

## Figures and Tables

**Table 1:** Summary of regulators’ role, relevance and main action.

Regulators	Role in the Pathway	Relevance in Bone/Dental Tissues	Main Action	Study Model	Ref.
GPC3 (Glypican-3)	Activator	Differentially expressed in dental pulp affected by caries	Can bind Wnt and Frizzled to enhance signaling depending on context	In vitro	[23,24,25]
Rspos (R-spondins)	Activator	Indirect relevance to alveolar bone and dental tissue regeneration	Potentiate Wnt/β-catenin signaling pathway by inhibiting Znrf3/Rnf43E3 ligases	In vitro/In vivo	[26,30,31]
CK (Casein Kinase)	Dual role context-dependent	CKIIP-1 identified as a negative regulator of MSC osteogenesis; limited data in dental development	Phosphorylates pathway components or promotes β-catenin degradation	In vitro	[32,35]
Norrin	Activator	Evidence not reported	Binds Frizzled-4 and LRP5/6 to activate β-catenin signaling in retina	In vitro	[36,38]
DKK	Inhibitor	Inhibit bone formation	Binds LRP5/6 and blocks Wnt–Frizzled–LRP complex formation	In vitro/in vivo	[39,40,42]
sFRPs (Secreted Frizzled Related-Proteins)	Dual role context-dependent	sFRP5 protective in periodontal tissues; sFRP3 promotes and sFRP4 inhibits osteogenesis in hMSCs	Bind Wnt ligands extracellularly, preventing receptor interaction; can upregulate the stabilization of β-catenin	In vitro/in vivo	[43,45,46]
WIF (Wnt Inhibitor Factor)	Inhibitor	Potential influence on bone-related dental processes	Directly binds Wnt proteins to inhibit their signaling	In vitro	[50,51]
Cardamonin	Inhibitor	Modulates bone remodeling	Suppresses Wnt/β-catenin signaling pathway via inhibition of nuclear β-catenin accumulation	In vitro/in vivo	[52,53]
SOST (Sclerostin)	Inhibitor	Potential impact on alveolar bone density and remodeling	Binds LRP5/6, preventing Wnt-induced receptor activation	In vitro/in vivo	[55,57]

## Data Availability

No new data were created or analyzed in this study. Data sharing is not applicable to this article.
